# Chitosan-Based Nanocomposites for Glyphosate Detection Using Surface Plasmon Resonance Sensor

**DOI:** 10.3390/s20205942

**Published:** 2020-10-21

**Authors:** Minh Huy Do, Brigitte Dubreuil, Jérôme Peydecastaing, Guadalupe Vaca-Medina, Tran-Thi Nhu-Trang, Nicole Jaffrezic-Renault, Philippe Behra

**Affiliations:** 1 Laboratoire de Chimie Agro-industrielle, LCA, Université de Toulouse, INRAE, 31030 Toulouse CEDEX 4, France; huyminh.do@ensiacet.fr (M.H.D.); brigitte.dubreuil@ensiacet.fr (B.D.); jerome.peydecastaing@ensiacet.fr (J.P.); guadalupe.vacamedina@ensiacet.fr (G.V.-M.); 2 “Water–Environment–Oceanography” Department, University of Science and Technology of Hanoi (USTH), Vietnam Academy of Science and Technology (VAST), 100000 Hanoi, Vietnam; 3 Centre d’Application et de Traitement des Agroressources (CATAR), Université de Toulouse, 31030 Toulouse CEDEX 4, France; 4 Faculty of Environmental and Food Engineering, Nguyen Tat Thanh University (NTTU), 700000 Ho Chi Minh, Vietnam; ttntrang@ntt.edu.vn; 5 Institute of Analytical Sciences, UMR 5280 CNRS-Université Claude Bernard, 69100 Villeurbanne, France; nicole.jaffrezic@isa-lyon.fr

**Keywords:** pesticides, glyphosate, surface plasmon resonance, chitosan, nanocomposites, ZnO, graphene oxide

## Abstract

This article describes an optical method based on the association of surface plasmon resonance (SPR) with chitosan (CS) film and its nanocomposites, including zinc oxide (ZnO) or graphene oxide (GO) for glyphosate detection. CS and CS/ZnO or CS/GO thin films were deposited on an Au chip using the spin coating technique. The characterization, morphology, and composition of these films were performed by Fourier-transform infrared spectroscopy (FTIR), atomic force microscopy (AFM), and contact angle technique. Sensor preparation conditions including the cross-linking and mobile phase (pH and salinity) were investigated and thoroughly optimized. Results showed that the CS/ZnO thin-film composite provides the highest sensitivity for glyphosate sensing with a low detection limit of 8 nM and with high reproducibility. From the Langmuir-type adsorption model and the effect of ionic strength, the adsorption mechanisms of glyphosate could be controlled by electrostatic and steric interaction with possible formation of 1:1 outer-sphere surface complexes. The selectivity of the optical method was investigated with respect to the sorption of glyphosate metabolite (aminomethylphosphonic acid) (AMPA), glufosinate, and one of the glufonisate metabolites (3-methyl-phosphinico-propionic acid) (MPPA). Results showed that the SPR sensor offers a very good selectivity for glyphosate, but the competition of other molecules could still occur in aqueous systems.

## 1. Introduction

**Figure 1:**
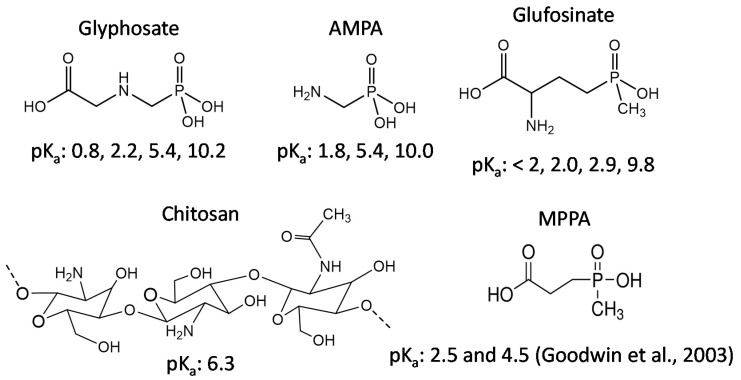
Structures and pK_a_ of glyphosate, chitosan (CS), and other studied compounds (molar mass of glyphosate: 169.07 g/mol; aminomethylphosphonic acid (AMPA): 111.04 g/mol; glufosinate: 181.13 g/mol; 3-methyl-phosphinico-propionic acid (MPPA): 152.09 g/mol) [40].

Glyphosate (N-(phosphonomethyl)glycine), the world most widely applied herbicide in agriculture and urban areas [1], was first synthesized by the Swiss chemist Henri Martin in 1950 [2] as a potential pharmaceutical compound [3]. A phosphonomethyl derivative of the amino acid glycine (Figure 1) [4], glyphosate is very polar, highly soluble in water (12 g/L or 71 mM at 25 °C) and insoluble in non-polar organic solvents (acetone, ethanol, and xylene) [5,6,7,8,9]. Depending on pH, glyphosate forms cationic and anionic sites within its structure [10], having a zwitterionic behavior from pH 1 to 10 [11]. The herbicidal activities of glyphosate were discovered in 1970 by John Franz and his co-workers at Monsanto [12]. Worldwide use of glyphosate in agriculture rose 13.3-fold, from 43 million kg in 1994 to 747 million kg in 2014 [2]. Currently, glyphosate is used in more than 130 countries, with a total global consumption estimated at over 825 million kg [13,14]. Glyphosate is widely used in agriculture because of its ability to control perennial weed species, overwintering rhizomes and tubers, its ability to bind to soil colloids, and to introduce transgenic, glyphosate-resistant crops [15,16]. Under environmental conditions, glyphosate can be quickly degraded to aminomethylphosphonic acid (AMPA), the main metabolite, and glyoxylate by microorganisms, physical and chemical degradation, or photodegradation [17,18,19]. As previously mentioned about the heavy agricultural use, glyphosate can accumulate in the environment, migrate into soil and aquatic system, and be detected in plant products [20,21,22,23].

There are reports presenting evidence of human exposure to glyphosate [24,25]; and some even addressing the impact of the glyphosate residues on human health. In this context, a correlation was established between the increased glyphosate use and various human diseases such as kidney damage, attention-deficit, hyperactivity disorder (ADHD), autism, Alzheimer’s and Parkinson’s diseases [26]. Literature also indicates that glyphosate may contribute to the development of cancer in humans and animals [27]. Different international agencies including the European Food Safety Authority (EFSA), the International Agency for Research on Cancer (IARC), or the US Environmental Protection Agency (US EPA), have evaluated the carcinogenicity of glyphosate [28]. In March 2015, an assessment by IARC concluded that glyphosate is probably carcinogenic to humans (group 2A) [29]. Subsequently, the development of an analytical method to detect glyphosate proved to be crucial, and that in order to control harmful pollutants in various environments such as surface and ground water, agricultural systems, or soil.

Given the properties above-mentioned, maximum residue thresholds of glyphosate are established at the very low concentration especially for drinking water (0.1 μg/L or 0.6 nM, in European countries) [30]. Liquid chromatography and gas chromatography coupled to mass spectrometry are traditional methods for glyphosate analysis [31,32,33]. However, the ionic and water-soluble proprieties of glyphosate make the analysis by HPLC advantageous over GC [34,35]. Capillary electrophoresis, coupled with mass electrophoresis was also implemented for the sensitive detection of glyphosate [36]. Although these are high sensitivity and selectivity methods, they present major drawbacks: complex behavior, specialized equipment, high operating costs, and time. To counteract the shortcomings of traditional analytical methods, developing a fast, simple, and low-cost method for glyphosate detection is a must. Noori et al. investigated an electrochemical sensor for glyphosate detection using Au electrode for amperometric measurements [37]. This method was successful in direct detection of glyphosate in drinking water without sample preparation or electrode modification, with a limit of detection (LOD) of 2 µM. In 2019, Mirmohseni et al. developed a sensitive layer based on a mixture of polydimethylsiloxane and acrylic polymer, which was coated on the surface with Au-coated quartz crystal chips for glyphosate detection using a quartz crystal nanobalance [38]. The calibration was obtained in the range of 0.2–8.6 mg/L (1.2–51 µM) with a LOD of 1.28 mg/L (7.6 µM). Recently, reports were published on a sub-nanomolar glyphosate optical sensor [39]. It is based on the functionalization of particle with enzyme, which can complex the analyte.

The surface plasmon resonance (SPR) seems very promising for glyphosate detection with potential benefits such as easy operation, high sensitivity, and reliability. From the Kretschmann’s configuration, the surface plasmon is obtained when an electromagnetic source interacts with free electrons on the interface between the thin metallic film and the dielectric media, which leads to the creation of an electromagnetic wave, propagating along the surface of the thin metal layer [41,42,43]. It is important to note that SPR only occurs when the momentum of the incoming light is equal to the momentum of the plasmon [41]. Such occurrence strongly depends on the changes of the refractive index near the Au-coated surface namely on the binding of molecules at the surface [44]. The SPR technique has received great attention in the field of sensor development for environmental monitoring (gas sensor, screening of trace elements or pesticides) [41,45,46,47]. The low-cost SPR instrumentation, based on fiber-optic, is still under development [48,49], and would be used for the on-field detection. The combination of active layers such as polyethylene, MoS_2_, and CS with SPR is capitalized on for sensitive detection of small molecules [46,49,50].

Chitosan ((1-4)-linked 2 acetamido-2-deoxy-β-D-glucan) (CS) is a linear cationic polysaccharide mainly produced by alkaline N-deacetylation of chitin shell from shrimps and other crustaceans [51,52]. CS offers many special characteristics including good film-forming, biodegradability, biocompatibility, low cost, non-toxicity, and good absorption properties. Due to its amine and hydroxyl groups, CS is suitable in potential applications for wastewater treatment, selective separation, and sensor development [53,54]. A process of cross-linking is typically applied to ensure the stable effectiveness of CS for the sorption and regeneration process. The selection of the type of the cross-linker and the reaction conditions can target the active site and the rate, respectively. The epichlorohydrin (EPI) is a cross-linking agent of OH groups of CS, while glutaraldehyde (GA) is reactive with primary amine functional groups [55,56,57]. CS is considered as a promising material for SPR sensor development to be applied in various fields [46,58].

The scope of this study is the capability of CS thin film as a probe for sensitive and selective glyphosate detection by SPR for further on-field detection. Graphene oxide (GO) and zinc oxide (ZnO) nanoparticles have excellent electrical and optical properties. They were hence selected for incorporation into CS to improve the sensitivity and selectivity of glyphosate sensor [59,60,61,62]. EPI and GA were used as cross-linkers under different conditions. Various parameters involved in the glyphosate detection were investigated and optimized to enhance the sensor selectivity. The SPR sensor selectivity was evaluated by comparing its behavior to other compounds containing similar structure and functional groups such as AMPA, glufosinate, and 3-methyl-phosphinico-propionic acid (MPPA), one of the glufosinate metabolites (Figure 1) [40,42].

## 2. Kinetic and Equilibrium Interaction Models

Different types of models can be used to describe interactions between glyphosate and the CS/ZnO sensing layer such as surface complex model [63,64]. Overall sorption reaction can be written as:

(1) $$>S+\mathrm{nA}⇆>{\mathrm{SA}}_{n}\;K_{\mathrm{LF},n}=\frac{\left\{>{\mathrm{SA}}_{n}\right\}}{\left\{>S\right\}\;{\left|A\right|}^{n}}$$

where {>S} represents the concentration (mol/g) of the surface sites, which can be either amine groups of CS or hydroxyl groups of CS or ZnO, |A| the activity of the aqueous molecule interacting with the surface, and $K_{\mathrm{LF},n}$ the thermodynamic constant.

Originally, this general model was developed to study the equilibrium for reversible sorption at solid-gas interfaces [64].

This general model has been extended in solution by taking into account the surface reactivity, such as surface acidity, surface complex formation or ion exchange, and aqueous speciation at equilibrium (see for more details Sigg et al., Charrière et al. [64,65]). From this model, different relationships, such as the Langmuir-type, Freundlich-type and Langmuir-Freundlich-type ones, can be deduced. They have been very often used for fitting reversible interactions at solid-liquid interfaces, at equilibrium.

The Langmuir-type model corresponds to the case of n = 1, by assuming homogeneous binding surface sites without any attraction or repulsion force, for which one monolayer can be formed. For example, we can consider the following reaction for a 1:1 surface complex (charges are not written):

(2) $$>S-\mathrm{XH}+A⇆>S-\mathrm{XA}\;+H\;K_{\mathrm{LF},1}=K_{L}=\;\frac{\left\{>S-\mathrm{XA}\right\}\;\left|H\right|\;}{\left\{>S-\mathrm{XH}\right\}\;\left|A\right|}$$

In that case, K_L_, the Langmuir-type coefficient depends on the acidity constants, the proton activity, the surface complexation constants, and the aqueous speciation of the sorbate, e.g., glyphosate (for more information, see Sigg et al., 2014 [64]).

If n ≠ 1, the Langmuir-Freundlich-type model corresponds either to the case of a reaction, for which n A molecules can react with one surface site or to heterogeneous surface behavior over the entire concentration range up to surface site saturation [66]. The Freundlich-type model refers to heterogeneous distribution or to the Langmuir-Freundlich-type model for which K_LF,n_ [C] << 1.

A pseudo-first-order kinetic relationship corresponding to relation (3) and the three different equilibrium isotherm models, Langmuir-type (4), Freundlich-type (5) and Langmuir-Freundlich-type (6) ones, were applied to the data of SPR sensor at constant pH value fixed [63,64,65,67,68,69]:

(3) $$\frac{d\Delta R}{dt}=k_{a}\left[C\right]\left(\Delta R_{max}-\Delta R\right)-{\;k}_{d}\Delta R\;K_{A}=\frac{k_{a}}{k_{d}}$$

(4) $$\Delta R=\frac{\Delta R_{max}K_{L}\left[C\right]}{\left(1+K_{L}\left[C\right]\right)}$$

(5) $$\Delta R=K_{F}{\left[C\right]}^{n}$$

(6) $$\Delta R=\frac{\Delta R_{max}K_{\mathrm{LF},n}{\left[C\right]}^{n}}{\left(1+K_{\mathrm{LF},n}{\left[C\right]}^{n}\right)}$$

with Δ*R*: variation in SPR angle (mdeg); Δ*R_max_* the maximum of the change in SPR angle (mdeg); dΔ*R*/*dt* the rate of change of the SPR signal; [*C*] the glyphosate concentration (M); k_a_ (M^−1^ s^−1^) and k_d_ (s^−1^) values of binding and dissociation rate constants, respectively; K_A_ (M^−1^) the forward coefficient at equilibrium, respectively; K_L_ the Langmuir-type coefficient (M^−1^); K_F_ the Freundlich-type coefficient (mdeg M^−n^); K_LF,n_ the Langmuir-Freundlich-type coefficient (M^−n^); n: the “heterogeneity” index.

## 3. Material and Methods

### 3.1. Chemicals and Instrumentation

CS (30000 g/mol, degree of deacetylation >90%) was obtained from Glentham Life Sciences (UK). ZnO (>97%, <50 nm), GO (2 mg/mL, dispersion in H_2_O), glyphosate (analytical standard), glufosinate (analytical standard), AMPA (analytical standard), EPI (analytical standard), GA (25%), acetic acid (≥99%), H_2_O_2_ solution (30%), NH_4_OH (99.98%), NaCl (≥99.5%), HCl (37%), and NaOH (>98%) were purchased from Sigma-Aldrich (France). All reagents were used without further purification. Ultra-pure water (UPW) was used for all solution preparation (18.2 MΩ.cm at 25 °C).

The Au sensor slides called later Au chips (SPR102-AU, 12 mm × 20 mm, BioNavis Ltd., Tampere, Finland) were taken from Bionavis™.

The polymer film was coated on the Au chip with a SPIN150i spin coater (POLOS spin coaters, Ingoldstadt, Germany).

The real-time detection of glyphosate in aqueous solution was carried out by MP-SPR Navi™ 200 OTSO (BioNavis Ltd., Tampere, Finland) using independent wavelength measurements (670 and 785 nm) with fluidic channels (standard flow-cell SPR Navi 200, SPR301).

FTIR spectra were measured at room temperature using a FTIR spectrometer (Spectrum 400, Perkin Elmer, Waltham, MA, USA) coupled to an attenuated total reflectance sampling accessory of diamond contact crystal. Recordings were obtained with a spectral width between 600 and 4000 cm^−1^ at a resolution of 4 cm^−1^.

AFM was used to investigate the surface morphology of the polymer films. Measurements were carried out using a Smart SPM-TM-1000 (AIST-NT, Novato, CA, USA) at Laboratoire de Chimie de Coordination (LCC, Toulouse, France). A MikroMasch tip HQNSC15-ALBs (silicon with an aluminum coating) was measured in tapping mode within a 5 × 5 μm sample area at a frequency of 265–400 kHz and a force constant K = 40 N/m.

The contact angles of the polymer films were recorded using a Tracker™ Automatic Drop Tensiometer (TECLIS Scientific, Civrieux d’Azergues, France). Measurements were carried out in accordance with the static “sessile drop” mode, using UPW as liquid phase (8.0 μL drops, n = 3), and then, contact angle values were measured for each sample.

### 3.2. Preparation of Thin Film

The Au layer of the chip was cleaned before use to ensure the stability and reproducibility of measurements made with the SPR instruments. An Au chip was placed in the boiling solution containing NH_4_OH (30%), H_2_O_2_ (30%), and UPW prepared with a ratio of 1:1:5 (v:v:v) at 80–90 °C. After 15 min, the chip was removed from the solution and immediately rinsed with UPW. Afterward, it was dried under a stream of N_2_, and then the slide was dried in air. Finally, the Au chip was treated by UV-O_3_ for about 10 min. Experiments were started right after this treatment.

**Figure 2:**
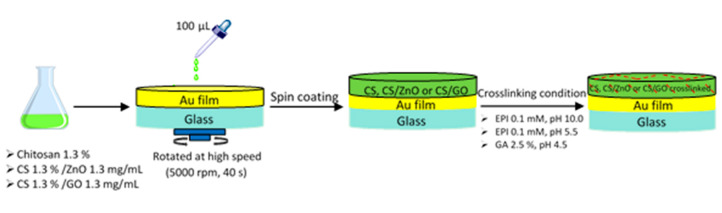
Schema of the preparation procedure for SPR sensor with different experimental conditions.

Preparation of 1.3% CS solution and SPR chip is summarized in Figure 2. Each CS nanocomposite solution was placed in an ultrasonic bath for 30 min. After spin coating, the cross-linking process was set to proceed under different conditions, followed by washing with UPW. FTIR, AFM and contact angles were used to investigate the characterization of the sensor surface.

### 3.3. Procedure of the Analysis

The concept of SPR sensor for the real-time determination of glyphosate was performed with four simultaneous measurements in two fluidic channels.

All measurements were carried out in a flow-through mode with a flow rate of 40 µL/min at 20.3 °C. The dead volume was estimated at around 97 µL, which corresponds to around 156 s between injection and the cell output. After inserting the SPR sensor into BioNavis system, a solution of 1 mM HCl was injected into the cell to wash the sensor surface. The sensor was stabilized in UPW after having reached a stable baseline. The UPW signal was recorded as a blank. Glyphosate solutions were then injected into the SPR system. The SPR signals vs. time at different glyphosate concentrations were recorded. Once the SPR signal reached a steady-state, the sensor was washed and stabilized to obtain a new baseline. Stabilization-sorption-washing steps were repeated for each glyphosate sample. The weighted centroid function of the SPR position (angle) was used to quantitatively determine the glyphosate sorption. Measurements performed at 670 nm showed a higher sensitivity than at 785 nm, hence the selection of this wavelength for qualification.

### 3.4. Evaluation of CS/ZnO SPR Sensor Response

In order to check the method reproducibility, three different SPR sensors were fabricated in the same conditions. The aqueous solution of glyphosate (0.30 μM) was then applied to three sensors. The SPR responses were used to evaluate the reproducibility of the SPR sensors.

To evaluate the reusability of the glyphosate SPR sensor, three stabilization-sorption-extraction cycles were repeated on the same SPR sensor with the aqueous solution of glyphosate (0.06 μM).

Limit of detection (LOD) and limit of quantification (LOQ) were estimated under the optimized conditions according to the relationships [70]:

(7) $$LOD=\frac{3\;s_{blank}}{slope}$$

(8) $$LOQ=\frac{10\;s_{blank}}{slope}$$

where *s_blank_* is the standard deviation of the blank measurements in the absence of glyphosate, and the slope corresponds to the regression line between SPR signal and glyphosate concentration over the linear range.

### 3.5. Statistical Analysis

The descriptive statistic was used to describe the basic features of the data in the study. All data presented in this work are the mean of three different SPR sensors, and the standard deviation was calculated as the square root of the variance of the three responses.

## 4. Results and Discussion

### 4.1. Characterization of CS-Based Nanocomposite Films

**Figure 3:**
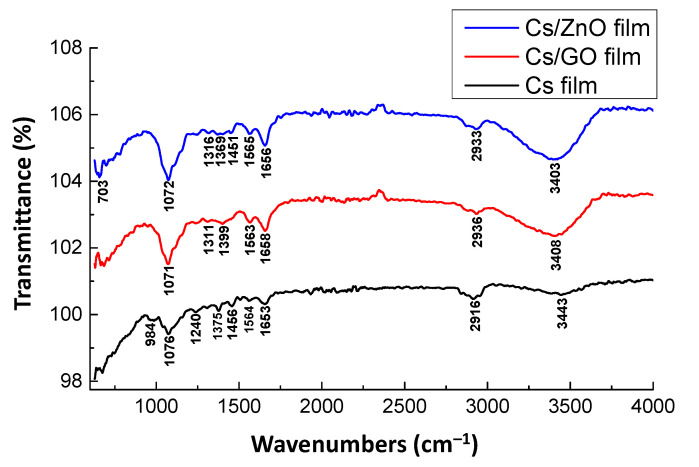
FTIR spectra of CS, CS/GO, and CS/ZnO composite thin film cross-linked by GA.

The thin films of CS, CS/GO, and CS/ZnO composite deposited onto the Au chips and cross-linked by GA were characterized by FTIR (Figure 3). The FTIR spectrum of CS film showed the main absorption bands at 3447 cm^−1^ assigned to the stretching vibrations of –N–H and O–H bonds [71,72] corresponding to free, intra-, and inter-molecular H-bonded groups (alcohol, amine, and amide). The vibrations related to the stretching of the bond C–H of –CH, –CH_2_, and –CH_3_ groups of CS could be characterized by the band in the region of 2916 cm^−1^ [73]. In the range between 1700 and 1500 cm^−1^, the FTIR spectra revealed the vibrations of several bonds. The vibration intensity and position depend on the cross-linking rate and the acetylation degree of CS.

The band close to 1653 cm^–1^ can be attributed to the stretching vibration of the C=O of the amide group [71,72] and of the imine bond (C = N) formed by a reaction between GA and amino groups. The absorption peak near 1564 cm^–1^ corresponds to the bending vibration of –N–H bond from both primary amine and secondary amine groups [71,72]. The peak shifts depending on the ratio of free primary amine (not cross-linked by GA) site to amide group. The –N–H binding vibration of primary amine group of CS is normally centered at 1595 cm^–1^ and those of amide group of chitin are observed at 1565 cm^–1^ [74].

The wide band centered at 1076 cm^−1^ can be assigned to the vibrations: C–O–C, C–OH, and C–CH_2_ stretching and CH, CH_2_, and CH_3_ bending [75]. The band at 984 cm^−1^ is associated to the aliphatic amine and the stretching vibration of CS ring [76]. In the case of CS/GO and CS/ZnO composite thin film, the presence of the most typical absorption peaks of CS was also observed in the FTIR spectrum of the composites. However, the peak in the range of 3400 cm^−1^ assigned to the stretching vibration of –OH and –NH_2_ groups shifted to a lower position and became broader and of higher intensity, indicating a strong interaction between these groups and the oxides (GO and ZnO). The characteristic bands of the C–H symmetric and antisymmetric stretching vibrations of CH_2_ and CH_3_ groups were shifted to higher wavenumber of 2933 and 2936 cm^−1^ for composites, which indicates a slight change of the functional group ratio due to the presence of oxides. Similarly, the vibration of C = O and C = N at 1653 cm^−1^ was shifted toward higher wavenumber and increased in the presence of nano ZnO or GO, which confirms that the amine and amide groups of CS were involved in the formation of linkage between CS and GO or ZnO. These FTIR results show the successful formation of composites between CS and GO or CS and ZnO.

**Figure 4:**
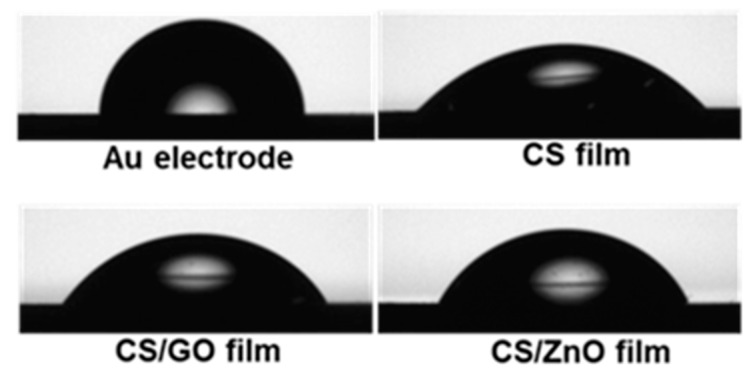
Contact angle measurements on Au electrode, CS, CS/GO, and CS/ZnO composite thin film cross-linked by GA.

The hydrophobic/hydrophilic nature of chips was evaluated by the contact angle technique at ambient conditions. Contact angle values for Au, CS, CS/GO, and CS/ZnO composite thin films are 91.2 ± 1.9, 47.3 ± 1.6, 60.9 ± 1.8, and 62.0 ± 0.6, respectively (Figure 4). Contact angle values decreased after coating by CS and its nanocomposites due to CS amino and hydroxyl, hydrophilic functional groups, indicating that hydrophilic films on the Au chip were formed. The increase in the contact angle value of CS nanocomposites compared to CS indicates that the attachment of GO or ZnO causes an increment in the hydrophobic properties of films. These results show that the CS, CS/GO, and CS/ZnO film could be formed on the Au chip.

**Figure 5:**
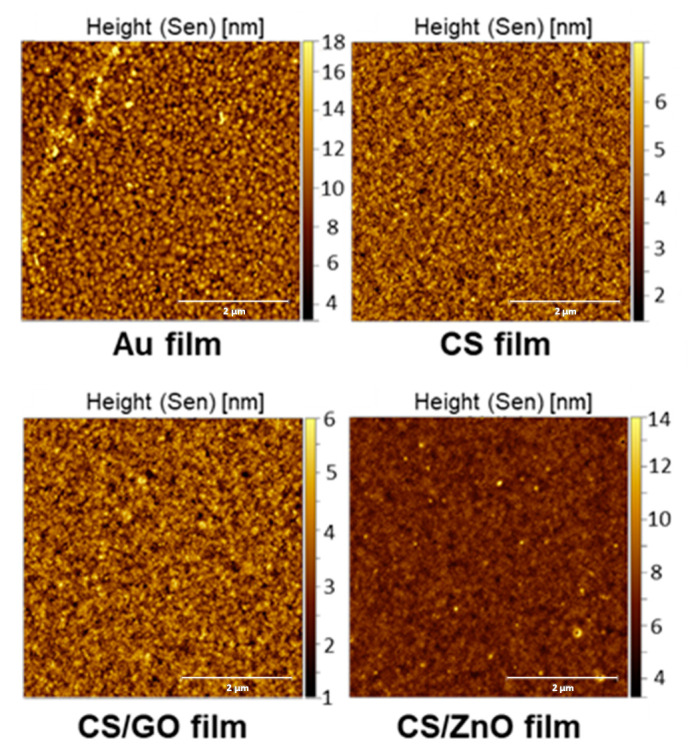
AFM images of Au electrode, CS, CS/GO, and CS/ZnO composite thin films cross-linked by GA.

Surface morphologies of various modified chips with CS, CS/GO, and CS/ZnO composite thin films were investigated by AFM (Figure 5). The roughness of the surface was measured using AFM analysis in the root-mean-square (RMS). The Au chip considered as reference was rough with a calculated RMS value of 1.85 nm. The AFM pictures showed a relatively smooth, compact, and homogeneous surface after coating with CS and its nanocomposites (Figure 5). The RMS values of CS, CS/GO, and CS/ZnO composite thin films were 0.946, 0.747, and 0.853 nm, respectively. In addition to that, very small bright spots corresponding to ZnO nanoparticles in the case of CS/ZnO film could be observed. These changes confirm that the CS and its nanocomposites film could be successfully achieved on the Au chips.

### 4.2. Sensitivity of CS-Based Nanocomposite Films for Glyphosate Detection

**Figure 6:**
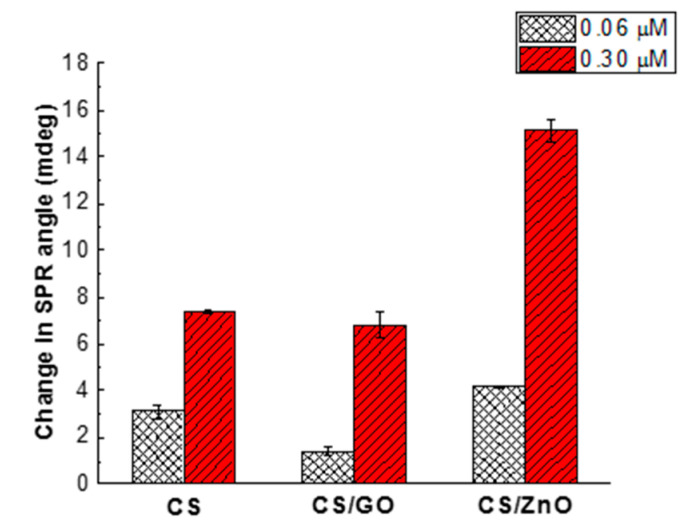
Sensitivity of glyphosate sorption via a change in angle on CS film alone, CS/GO, and CS/ZnO film cross-linked by GA for two glyphosate concentrations (0.06 and 0.30 μM), at pH 5.5.

The experiments were performed to investigate the CS-based composites for enhancing the sensitivity and selectivity for two glyphosate concentrations, 0.06 and 0.30 μM, at pH 5.5 (Figure 6). The highest increase in the SPR angle change was recorded by CS/ZnO SPR sensor compared to CS (2 times) and CS/GO (1.5 times) ones. With the CS SPR sensor, the sorption of glyphosate depended only on amine and hydroxyl groups of CS. The increase in the sorption for CS/ZnO SPR sensor could be explained by the complex formation between glyphosate and ZnO at the liquid-solid interface [77]. On the other hand, the incorporation of CS and ZnO nanoparticles was assumed to improve optical properties leading to enhance the sensitivity of CS/ZnO SPR sensor [62,78]. In the case of CS/GO SPR sensor, the optical properties of GO could not improve the glyphosate sorption because of the non-uniformity and cracks of the CS/GO composite coated on the Au chip. Consequently, the CS/ZnO SPR sensor was selected for further experiments.

### 4.3. Effect of Cross-Linking Conditions on the Sensitivity of CS/ZnO Composite Films

**Figure 7:**
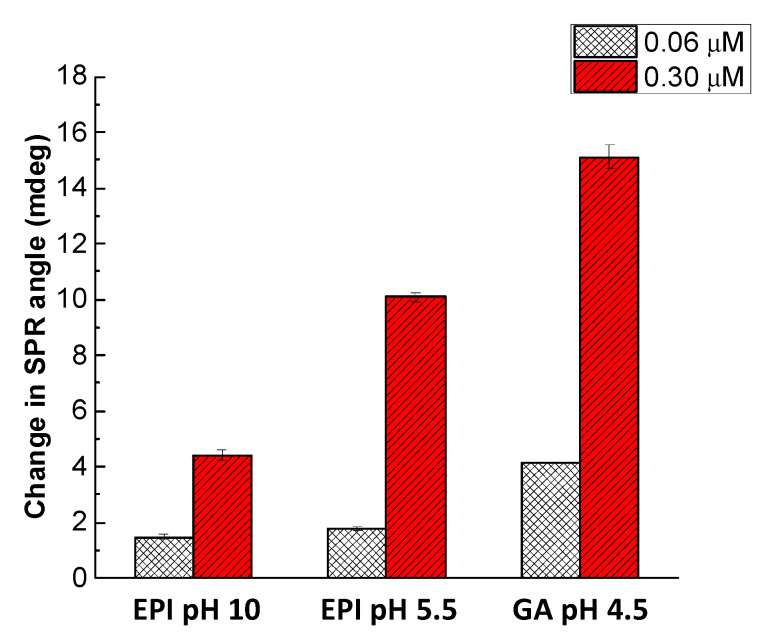
Glyphosate sorption onto CS/ZnO SPR sensor cross-linked for two glyphosate concentrations (0.06 and 0.30 μM), at pH 5.5. Experimental conditions: cross-linking for 2 h with GA (2.5%, pH 4.5), EPI (0.1 mM, pH 10) at 40 °C or EPI (0.1 mM, pH 5.5) at 40 °C followed by washing with UPW.

The sensitivity of three CS cross-linking conditions on the sorption capacity of the three SPR sensors was evaluated through SPR responses for two glyphosate solutions (0.06 and 0.30 μM) (Figure 7). Overall, CS/ZnO cross-linked in acidic conditions displayed a higher SPR response for glyphosate than the film cross-linked in alkaline conditions. The protonation of amino groups on cross-linked CS/ZnO thin film could explain it, since acidic conditions would increase the interactions between glyphosate and the receptor.

Results showed higher values of SPR response for GA cross-linked SPR sensor than other SPR sensors. Some studies have also shown that the adsorption capacity of the CS cross-linked by GA was favored by the presence of amino groups leading to the improvement of the electrostatic and hydrophobic properties of CS [79,80]. In the case of SPR sensor cross-linked by EPI in acidic medium, the lower SPR response could be explained by the fact that amino groups are partially blocked [79,81]. For that reason, GA was selected as a cross-linker for CS/ZnO composite thin film at pH 4.5.

### 4.4. Effect of pH on the Sorption Capacity of CS/ZnO Composite Films

In this study, the different solutions were not buffered after addition of HCl or NaOH. Buffering is a critical issue when studying the role of pH, for buffer addition is not neutral for various reasons as discussed by many authors [82,83]. The first reason is the role of electrolyte used, which can interfere with the target molecule and modify its behavior as observed by some authors [65,84,85]. Moreover, buffering can hide some exchange processes due to the constant pH [84,85]. The alternative would be to leave the pH free. In fact, different authors worked without buffer to avoid such drawback [86,87,88]. In this study, we decided to avoid such interactions by removing all buffering molecules.

**Figure 8:**
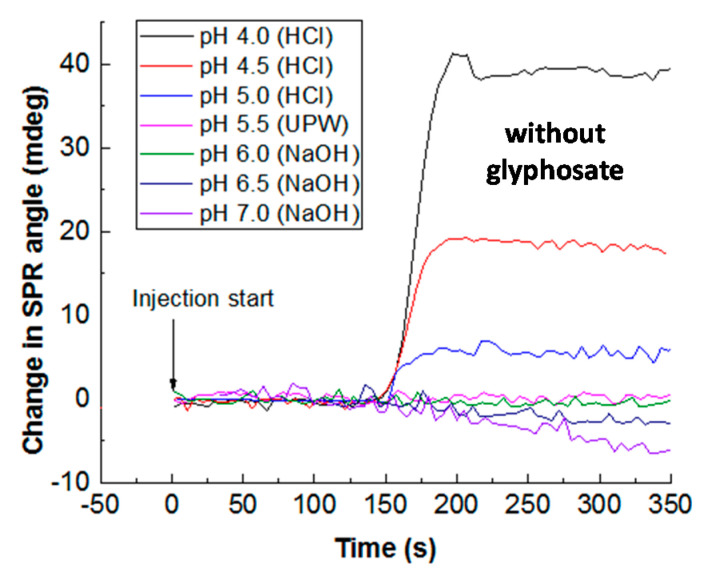
Role of pH without glyphosate in the change in SPR angle in the case of CS/ZnO film cross-linked by GA (dead time corresponds to around 156 s between injection and the cell output). Experimental conditions for each reported pH value: injection of UPW (pH 5.5) before injection of a solution at a given pH adjusted by 0.01 M HCl and 0.01 M NaOH into the SPR system.

As observed in Figure 8, the SPR response at steady state depends on pH, decreasing from pH 4.0 to 7.0, corresponding to a titration of the surface sites of CS/ZnO film cross-linked by GA.

**Figure 9:**
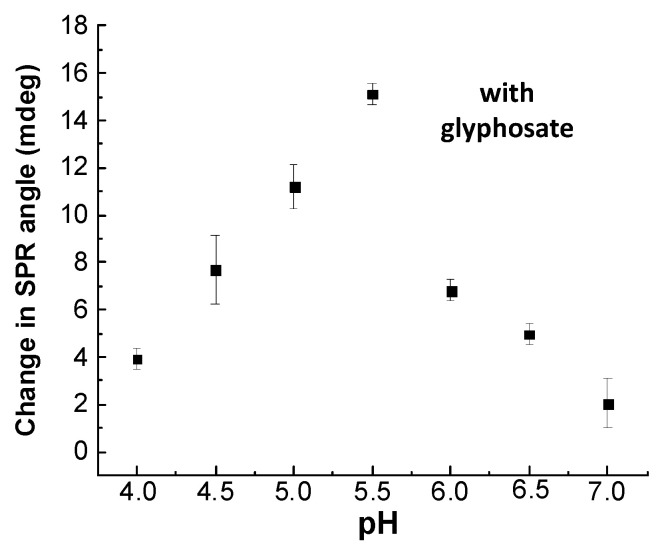
Role of pH (4.0 to 7.0) in the change in SPR angle in the case of CS/ZnO film cross-linked by GA in the presence of 0.30 μM glyphosate (dead time corresponds to around 156 s between injection and the cell output). Experimental conditions: (**i**) first, injection of a non-buffered solution at a given pH adjusted by 0.01 M HCl and 0.01 M NaOH without glyphosate into the SPR system to obtain a steady-state baseline, followed by the injection of the glyphosate solution at the same pH, and the change in SPR angle corresponding to the effect of glyphosate with respect to the baseline before glyphosate injection; (**ii**) same procedure at another pH value.

The sorption of glyphosate at 0.30 μM onto CS/ZnO adsorbents was valuated from pH 4.0 to 7.0 (Figure 9). The SPR response increased and then decreased after reaching a maximum pH value at 5.5. The interaction between the SPR sensor and glyphosate was controlled by the protonation/deprotonation of the CS and glyphosate [82]. The SPR sensor had lower sorption capacity when pH decreased from 5.5 to 4.0, due to the protonation of glyphosate (see Figure 8). Meanwhile, most of the free amine groups on the CS molecule were still protonated in acid medium (pH < pK_a_ = 6.3, for CS). When pH of the glyphosate solution increased from 5.5 to 7.0, the sorption amount of glyphosate on the SPR sensor significantly decreased for the SPR sensor, which could be explained by the deprotonation of amino groups. Moreover, aqueous OH^−^ groups could compete with glyphosate, negatively charged under alkaline condition [89,90]. According to the result, pH 5.5 was selected as the optimal pH in the following experiments.

### 4.5. CS/ZnO SPR Sensor Response for Glyphosate at pH 5.5 and Ionic Strength (I) Effect

**Figure 10:**
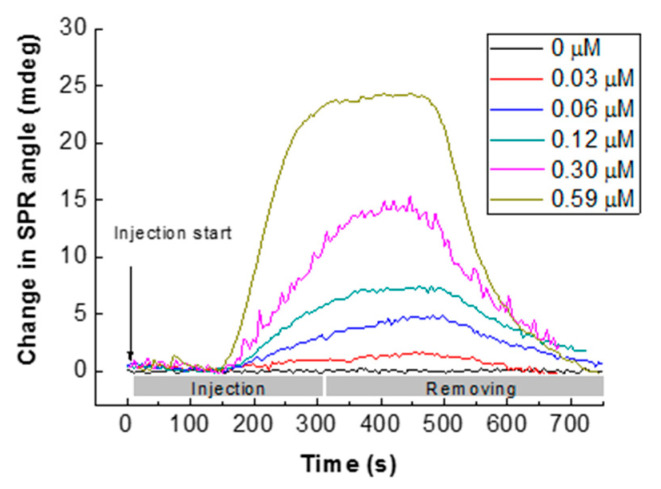
Typical sensorgrams of glyphosate sorption onto CS/ZnO SPR sensors for glyphosate concentration from 0 to 0.59 µM (dead time corresponds to around 156 s between injection and the cell output).

Real-time responses of the sensor vs. aqueous solutions of glyphosate at different concentrations are reported in Figure 10. The increase in glyphosate concentration led to an increase in SPR sensor response. SPR signals of the sensor increased quickly when the glyphosate solution reached the sensor surface. The changes in SPR response were monitored in real-time and reached a steady state at about 250 s before desorption, which was obtained after the injection of UPW at pH 5.5. The total desorption was obtained after more than 450 s.

**Figure 11:**
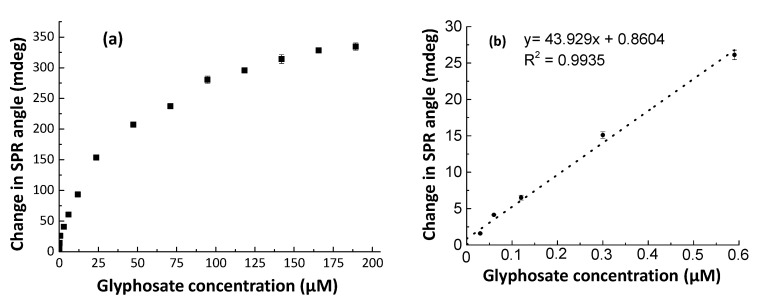
Glyphosate sorption onto CS/ZnO SPR sensors at pH 5.5: (**a**) full sorption isotherm of glyphosate; (**b**) calibration curve in the glyphosate concentration ranges between 0.03 and 0.59 μM.

Under optimal experimental conditions, CS/ZnO SPR sensors interacted with aqueous glyphosate solutions in the concentration range of 0–189 μM at pH 5.5 (Figure 11a).

The SPR response showed the reasonable linearity of the sensor response vs. glyphosate concentration estimated in the range of 0–0.59 μM with a regression coefficient (R^2^) value of 0.9935 (Figure 11b). In the investigated concentration range, the LOD and LOQ values of the CS/ZnO SPR sensor were calculated to be 0.008 and 0.027 μM, respectively.

The relative standard deviation (RSD) for three different CS/ZnO sensors was 5.2% at the glyphosate concentration of 0.30 μM, which corresponds to a good reproducibility. The RSD was about 4.6% for three stabilization-sorption-extraction cycles with the same sensor for the glyphosate solution of 0.06 μM, revealing thus the reusability of the sensor. Furthermore, the CS/ZnO sensor exhibited an excellent analytical performance in terms of sensitivities compared to previous studies related to optical methods for glyphosate detection (Table 1).

**Figure 12:**
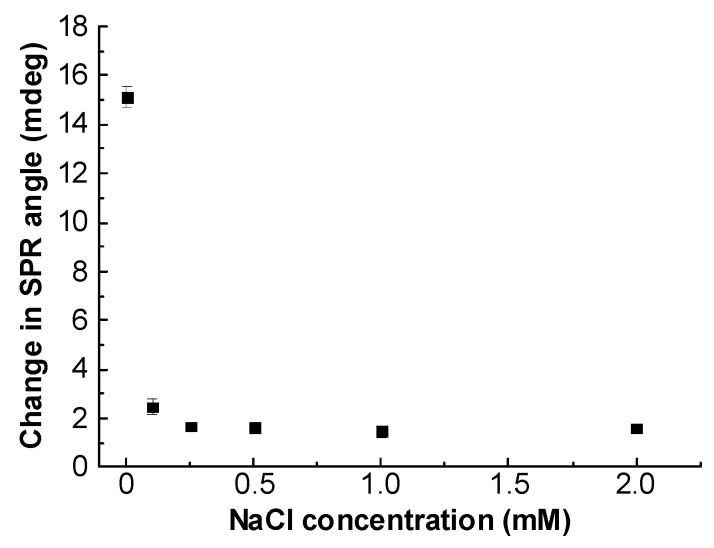
Effect of ionic strength (NaCl concentration) on the glyphosate sorption onto CS/ZnO SPR sensor at 0.30 μM glyphosate and pH 5.5.

The effect of ionic strength, I, (NaCl as electrolyte) on the glyphosate behavior in the presence of the CS/ZnO SPR sensor was investigated for I range from 0 to 2 mM at pH 5.5 (Figure 12). Overall, the SPR response strongly decreased with the increase in I. At higher I value, no such change was observed.

### 4.6. Sorption Mechanisms

CS/ZnO SPR responses were used to estimate kinetic and isotherm parameters based on Equations (2) to (5) from data reported in Figure 11 and Table 2. Parameters for the pseudo-first-order kinetics were estimated with a R_max_ value of 340 (mdeg) obtained from experimental data. The experiment showed that the estimated value of the binding rate constant k_a_ (1.9 × 10^3^ M^−1^.s^−1^) was higher than the value of the dissociation rate constant k_d_ (5.8 × 10^−4^ s^−1^). The estimated K_A_ value was very high meaning that the affinity between the CS/ZnO film and glyphosate was very strong for such experimental conditions.

The analysis of the isotherm models is reported in Table 2. Based on the regression coefficient (R^2^) value, the data fit well with all equilibrium isotherm models. The R_max_ values estimated from the Langmuir-type adsorption isotherm model (357 mdeg) were very close to the maximum experimental value (340 mdeg). In the investigated range of concentrations, it could be assumed that a 1:1 surface complex is formed as described by Reaction (6) between glyphosate and the available surface sites. Moreover, these results indicated that glyphosate binding sites are homogeneously distributed and that there is no interaction between adjacent adsorbed molecules [67].

From Figure 12 showing I effect, it can be concluded that outer-sphere surface complexes are formed in a first stage due to the change in the double-layer thickness and/or by competition between glyphosate and the counter-ions, Na^+^ and Cl^−^. In such conditions, the electrostatic interaction could control the glyphosate behavior at the CS/ZnO film surface.

### 4.7. Selectivity of CS/ZnO Composite Films

**Figure 13:**
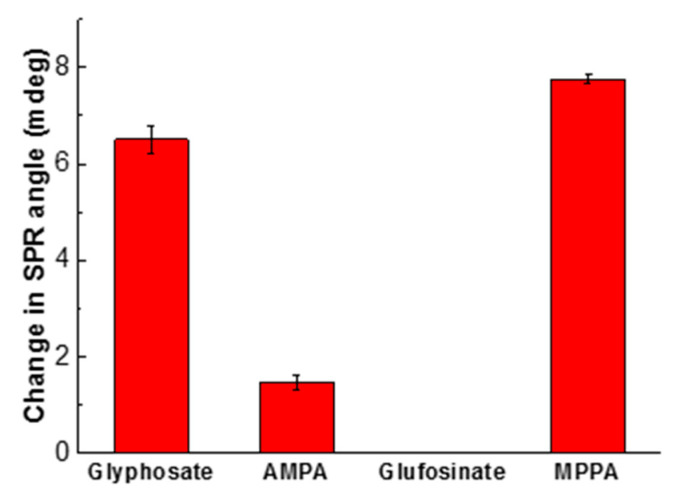
Sorption behavior of glyphosate, AMPA, glufosinate, and MPPA on CS/ZnO SPR sensor at 0.12 μM and pH 5.5. Experimental conditions: injection of each solution separately into the SPR system with a flow rate of 40 µL/min at 20.3 °C.

Since the selectivity is a very important property to test the practical application of the chemical sensor with respect to glyphosate, the studies were carried out under optimal conditions for glyphosate, AMPA, glufosinate, and MPPA, at the same concentration (0.12 μM) and pH 5.5 (Figure 13). Overall, the CS/ZnO SPR sensor response of glyphosate was 4.3 times higher than the one of AMPA, while the sensor was not sensitive to glufosinate. However, it appeared that the SPR response ratio between MPPA and glyphosate was around 1.15, indicating a better sorption of MPPA to the CS/ZnO than the glyphosate one.

At pH 5.5, the CS/ZnO layer is positively charged, AMPA and glufosinate are –1 negatively charged and MPPA and glyphosate –2 negatively charged. The different charges between AMPA and glufosinate could be due to the location of the OH groups, closer in the first one compared to the second one. On the other hand, the sorption of MPPA could be more favorable due to the absence of the positive charge in MPPA compared to glyphosate. These results are thus consistent with electrostatic effect and support the fact that sorption is reversible in our experimental conditions, which is critical for sensor development.

## 5. Conclusions

The SPR sensor based on CS and its nanocomposites for the real-time detection of glyphosate in the aquatic system was developed. In this study, different nanomaterials such as ZnO and GO were incorporated into CS to enhance the sensitivity and selectivity of the SPR sensor. The CS/ZnO SPR sensor exhibited a better sensitivity than CS and CS/GO SPR sensors. Different parameters (cross-linking condition and pH of sorption) were investigated and optimized for the SPR sensor based on CS/ZnO composite thin film. From the above results, the optical sensor for the detection of glyphosate using SPR and based on CS/ZnO was synthesized and characterized by different techniques such as AFM, FTIR, and contact angle technique. Under the optimal experimental conditions (cross-linking condition and pH on the sorption of glyphosate), the sensor showed a good linear response vs. glyphosate concentration (0–0.59 μM) with estimated LOD and LOQ of 0.008 μM and 0.027 μM, respectively. From isotherm models, the Langmuir-type one can be used to explain the glyphosate sorption behavior. Main mechanisms would be controlled by electrostatic and steric interaction with the possible formation of 1:1 outer-sphere surface complexes between glyphosate and surface sites. In addition, the SPR sensor showed a good selectivity when testing with AMPA and glufosinate, but a lower one in the presence of MPPA. Further work needs to be conducted to improve the selectivity with respect to other molecules such as organic ligands and the impact of some cations before applying this method for monitoring glyphosate in natural waters.

## Figures and Tables

**Table 1:** Comparison of the CS/ZnO sensor with other optical methods for glyphosate detection.

Sensor	Linear Range	LOD	Ref.
Colloidal Ag nanoparticles/SERS ^a^	237–350 µM	10 µM	[91]
AgNPs ^b^/SERS	-	5.3 µM	[92]
Cu doped poly(vinyl) alcohol)) nanofiber/colorimetric	0.59–2958 µM	0.59 µM	[93]
Carbon dot labelled antibodies/magnetic NPs/fluorescence	0.059–473 µM	0.047 µM	[94]
2-mercapto-5-nitrobenzimidazole capped AgNPs (MNBZ-AgNPs)/Mg^2+^/colorimetric	399–517 nM	17.1 nM	[95]
Hydrophobin-EPSPS ^c^ Fusion Protein/Spectrophotometer.	-	0.005 μM	[96]
CS/ZnO/SPR	0–0.59 μM	0.008 μM	This work

^a^: Surface-enhanced Raman spectroscopy; ^b^: Silver nanoparticles; ^c^: Enzyme: 5-enolpyruvylshikimate-3-phosphate synthase.

**Table 2:** Estimated parameters of kinetic law and sorption isotherm relationships in the case of glyphosate sorption onto CS/ZnO SPR sensors at pH 5.5 (see relationships (3) to (6)).

**Pseudo-First-Order Kinetics**									
k_a_ (M^−1^ s^−1^)		k_d_ (s^−1^)		K_A_ (M^−1^)		R^2^			
1.9 × 10^4^		5.8 × 10^−4^		3.3 × 10^7^		0.998			
**Equilibrium Isotherm Models**									
Langmuir-type			Freundlich-type			Langmuir–Freundlich-type			
ΔR_max_ (mdeg)	K_L_ (M^−1^)	R^2^	n	K_F_ (mdeg.M^−n^)	R^2^	ΔR_max_ (mdeg)	n	K_LF,n_ (M^−n^)	R^2^
399	2.5 × 10^4^	0.994	0.44	1.6 × 10^4^	0.991	550	0.71	2.7 × 10^5^	0.998
